# Regime-switching empirical similarity model: a comparison with baseline models

**DOI:** 10.1007/s00181-022-02214-8

**Published:** 2022-02-13

**Authors:** Jamol Bahromov

**Affiliations:** Ruhr-Universität Bochum, Universitätsstraße 150, 44801 Bochum, Germany

**Keywords:** Empirical similarity, Regime switching, ML estimation, Markov-switching autoregression

## Abstract

In this paper, I extend the standard specification of the empirical similarity (ES) model of Gilboa et al. (Rev Econ Stat 88:433–444, 2006) to account for changes in parameters. I implement this by allowing for a combination of component ES models in the spirit of Gaussian mixture models. The predictive power of the modified model, along with that of the standard specification, will be assessed and compared to the baseline models consisting of autoregressions and Markov-switching autoregressions within a simulation exercise. Finally, we also compare the predictive ability of models using data on quarterly US real GDP growth. The results indicate that in situations of a more complex regime-switching behavior and a moderate to high autocorrelation in series, modified ES model demonstrates a better empirical fit. In addition, results of the empirical example show that modified ES models can better predict more extreme observations.

## Introduction

The empirical similarity (ES) model entered the econometric research domain relatively recently. The theoretical framework of the model derives itself from the case-based decisions theory. The theory of case-based decisions (CBDT) refers to a set of axioms aimed at justification of analogy-based reasoning by humans in the face of uncertainty. CBDT is based on the idea that from similar causes one can expect similar effects. Gilboa and Schmeidler (1995), who formally developed the theory, argue that expected utility theory as a conventional model of human reasoning under uncertainty looses ground when the decision problem at hand is complicated in the sense that alternative states of the world and outcomes of actions associated with them cannot be exhaustively defined. In such situations, people choose acts based on their performance in similar problems they have experienced in the past (i.e., Gilboa and Schmeidler 1995; Gilboa et al. 2014).

ES is essentially an operationalization of the CBDT in the form of a statistical model. Unlike usual regression-based models which focus on defining explicit functional relations between the dependent and independent variables, the ES model is designed as a universal tool to generate predictions on the evaluation target. Assumption of a functional relationship between the dependent *Y* and an independent variable *X* is preceded by extensive theoretical analysis and followed by necessary ex post testing of hypotheses. In contrast, similarity-based averaging is argued to be a good first-order approximation of human reasoning in the situation of uncertainty of functional relationships. However, this does not imply that ES model is only applicable to data generated by human reasoning. Even though in reality the relation between *Y* and *X* is governed by a functional rule *f*, the ES technique can still be used to make predictions for *Y* as long as *f* is sufficiently smooth and the sample size is large (Gilboa et al. 2006).

The ES model specifies the value of the target variable to be weighted average of its previous values. This relates the model to the class of autoregressive models.^1^ Therefore, this paper endeavors to conduct a comparative study of ES model to the autoregressive and Markov-switching (MS) autoregressive models. Autoregressive model is the main candidate in predictive time series analysis to model the autocorrelation dynamics in a variable. However, these models are unable to address the possible nonlinearities, in which case their Markov-switching versions are employed. MS autoregressions attempt to accommodate the nonlinear behavior through changes in parameters. At each time point, the target variable is assumed to visit one of *K* different states where its dynamics are governed by the parameters specific to the prevalent state. Although MS autoregressions entail some flexibility, their potential to embrace more complex forms of nonlinearities is rather limited. In contrast, the ES model, instead of imposing a strict structure, assumes the new values of the target variable to be distributed around the weighted average of its history of realizations where more similar observations receive larger weighting. Despite this seemingly trivial specification, the model offers versatility to fit various data situations. In fact, even though the underlying data-generating process was of regime-switching type like in MS autoregressive models, similarity-based averaging mechanism of the ES model should treat observations from the same regime as most relevant to each other. Of course, one cannot guarantee that the model functions as claimed and rather see this as a desired property. Nevertheless, by extending the ES model to accommodate regime change one can improve the ability of the model to account for potential differences in the conditional expectation of data points.

The paper is structured as follows. Section 2 contains theoretical discussion consisting of three subsections. The first subsection discusses how ES model fits into time series framework. The second subsection presents the base specification of the model. The final subsection introduces the regime-switching ES model and discusses the estimation. Section 3 is dedicated to the simulation study. The final section provides a comparison of the ES and baseline models in an empirical context.

## Theoretical discussion

### Autoregression and ES model

I have already noted above similarity of the specifications of autoregressive models and the ES model. In this section, we revisit this statement and discuss further how ES model compares to established models in time series analysis and why it could compete with these models in terms of empirical power under a slight modification.

A workhorse of modeling the dynamics of a random series $\{y_t\}_{t=1}^T$ has been a linear autoregression of the form:

1 $$\begin{aligned} y_t=\mu + \sum \limits _{l=1}^{L}\alpha _l y_{t-l} + \epsilon _t, \epsilon _t\sim \mathcal {N}(0,\sigma ^2) \end{aligned}$$

where $\mu$ is a constant term, $\alpha$—an autoregressive coefficient, $\sigma ^2$—an error variance, and *L*—the lag order. In the next section, it will be clear that ES model can also be viewed as an autoregression with $L = t-1$. One of the major disadvantages of (1) is that in practice systems of variables are subject to structural changes rendering the constant coefficient autoregressions inappropriate to adequately describe the data. Thus, both univariate models and their multivariate versions have undergone significant modifications targeted at accommodating parameter instabilities.

To a large extent, subsequent research focused on an introduction of a mechanism into the DGP of (vector-) autoregressive models that would govern the shifts in parameters. Tong (1999) argued that nonlinearities in observed data can be captured reasonably well with locally linear models. Subsequently, he introduced the *threshold autoregressive models* (TAR) which were extended to multivariate series by Tsay (1994). The general form of TAR models was a slight modification to that of (1):

2 $$\begin{aligned} y_t= & {} \mu (k) + \sum \limits _{l=1}^{L}\alpha _l(k) y_{t-l} + \epsilon _t, \epsilon _t\sim \mathcal {N}(0,\sigma ^2(k)) \text {if} c_{k-1}<z_t \le c_{k},\nonumber \\ k= & {} 1,\dots ,K. \end{aligned}$$

where $z_t \in \mathbb {R}$ and sets $\mathbb {R}_k=\{z_t|c_{k-1}<z_t\le c_{k}\}$ are *K* partitions of the domain of $z_t$ such that $\bigcup \nolimits ^K_{k=1}\mathbb {R}_k=\mathbb {R}$. According to (2), model parameters assume values corresponding to a state *k* if $z_t\in \mathbb {R}_k$. If $z_t=y_{t-d}$ for some positive integer *d*, the model is known to be called *self-exciting threshold autoregressive model* (SETAR). This kind of models provides enough flexibility to approximate arbitrarily closely any given nonlinear dynamics (Lim and Tong 1996).

The specification of TAR model given in (2) implies an abrupt switch between regimes. Chan (1986), however, argued that in some cases it may be reasonable to assume rather a gradual regime change. In their comprehensive survey of major developments, Dijk et al. (2010) discuss a *smooth transition* TAR, the basic representation of which is given as

3 $$\begin{aligned} y_t= \phi '_1 x_t(1-G(z_t;\gamma ,c))+ \phi '_2 x_t G(z_t;\gamma ,c)+\varepsilon _t \end{aligned}$$

where $x_t=(1,\tilde{x}_t')'$ with $\tilde{x}_t'=(y_{t-1},\dots ,y_{t-L})'$ and $\phi =(\phi _{k,0},\phi _{k,1},\dots ,\phi _{k,L})'$, $k=1,2$. The transition function $G(z_t;\gamma ,c)$ is bounded between 0 and 1. Teräsvirta (1994) assumes the transition variable $z_t$ to be a lagged endogenous variable, such that $z_t=y_{t-d}$ for some integer $d>0$. However, Dijk et al. (2010) argue that it could also be an exogenous variable or some function of it.

Transition variable $z_t$ in models discussed above can be both observable and unobservable. Assumption of an unobservable transition variable led to the development of a subclass of regime-switching models based on *Markov chains*. The methodology of Markov-switching models (i.e., see Krolzig and Toro 1999) relies on combining the conditional distributions $f(y_t|\mathcal {S}_t=k,\mathcal {F}_{t-1};\varvec{\theta })$, where $\mathcal {S}$ is an unobservable discrete state variable and $\mathcal {F}_{t-1}$—an available information set, at each *t* according to a probability distribution over a finite number of values of $\mathcal {S}$ with view to maximize the sample marginal likelihood $\prod _{t=1}^{T}f(y_t|\mathcal {F}_{t-1};\varvec{\theta })$ with respect to the parameter vector $\varvec{\theta }$. The probability distribution of states is assumed to evolve according to a discrete Markovian chain, and *expectation maximization* algorithm is used to produce the best inference on the state probabilities at given *t*.

The models discussed above have been developed to account for a nonlinear behavior in a time series. Although regime-switching mechanism of these models captures some nonlinearity due to shifts in parameters, the models remain linear locally within each state. This results in a negligence of an intrinsic nonlinearity of a given process. In such cases, it might be worthwhile to consider models with more flexible structure with an ability to capture complexities in data as well as demonstrate a decent external validity. ES model would make a good candidate assuming $y_t$ has features $\varvec{x}^{(k)}_t=(x^{(k)}_{1,t},x^{(k)}_{2,t}\dots x^{(k)}_{M,t})'$ specific to each state $k \in \{1,2,\dots ,K\}$ and the data set is rich enough to contain information about all possible states. In theory, the case-based nature of the model should make sure that observations from the same state should be treated as most similar. Like in autoregression, the feature vector $\varvec{x}^{(k)}_t$ can include lagged values of $y_t$. As will be detailed in the following section, there are two specifications of the basic model: “ordered” and “unordered.”

The former keeps the natural ordering of observations such as time ordering, whereas the latter does not consider information about ordering. Namely, in the “ordered” version, observations $t+i$ with $i = -t+1,-t+2,\dots , T-t$ can all participate in prediction of the observation *t*, while only $i = 1,2,\dots ,t-1$ participate in predictions in case of the “ordered” model. While one can agree with the connection between the ordered ES and an autoregression, reconciliation of the unordered ES with an autoregression might seem implausible. Nevertheless, the set of our baseline models mostly consists of regime-switching autoregressions which during estimation procedure use filtering and smoothing algorithm to provide inference on conditional state probabilities. This algorithm as well exploits the information from $t+1$ to *T* to derive the inference about state probability for *t*.

Gilboa et al. (2006) argue that ES models with lagged values of the target variable as features are akin to defining “patterns” in series through lagged values and searching for similarity in patterns. However, in order for this to be valid, observations defining these patterns should not behave erratically. As mentioned earlier, we simulate data under DGPs with regime switching. Thus, a sequence of observations may be far from constituting a pattern if they come from different local models of the DGP. In this sense, the ES model might not be on a level playing field with the baseline regime-switching models. Therefore, after the introduction of the basic model, I describe the modified ES model that accounts for changes in the mean of a variable *y* and the rest of its dynamics are handled by similarity averaging.

### The base specification of the ES model

The ES model derives itself from the concept of case-based reasoning, and its DGP is specified as

4 $$\begin{aligned} y_t=\sum \limits _{i<t}\psi _{t,i} y_i + \varepsilon _t, \varepsilon _t{\mathop {\sim }\limits ^{i.i.d}} (0,\sigma ^2) \end{aligned}$$

where

5 $$\begin{aligned} \psi _{t,i}=\frac{s(\varvec{x}_t,\varvec{x}_i)}{\sum \nolimits _{i<t} s(\varvec{x}_t,\varvec{x}_i)} \end{aligned}$$

$s(\varvec{x}_t,\varvec{x}_i)$ is a function $s:\mathbb {R}^n \times \mathbb {R}^n \rightarrow \mathbb {R}_{[0:\infty )}$ that turns features $\varvec{x}\in \mathbb {R}^n$ into the measure of similarity of values of *y* between *t* and *i*. The relation $i<t$ under the sum operator in (4) indicates that a value of *y* at *t* is a weighted average of preceding $t-1$ values. Gayer et al. (2007) later introduced the version of the model where $i\ne t$, where a given value is a weighted average of the rest of the sample. The functional form for $s(\varvec{x}_t,\varvec{x}_i)$ is usually chosen to be

6 $$\begin{aligned} s(\varvec{x}_t,\varvec{x}_i)=\exp (-(\varvec{x}_t-\varvec{x}_i)'\varvec{w}(\varvec{x}_t-\varvec{x}_i)) \end{aligned}$$

where $\varvec{w}=diag\{w_m\}_{m\le M}$. One can collect $\psi _{t,i}$ for all *t* and *i* into a $T\times T$ matrix $\varvec{C}$, where *T* is the length of data, $y_t$ and $\varepsilon _t$ into $T\times 1$ vectors for all *t* and rewrite (4) as

7 $$\begin{aligned} \varvec{y}=\varvec{C}\varvec{y}+\varvec{\varepsilon }. \end{aligned}$$

For $i<t$, $\varvec{C}$ is a lower triangular matrix, whereas for $i\ne t$ it is a full matrix. In both cases, its main diagonal consists of 0’s, so that there is not an identification issue for elements of $\varvec{C}$.

Equation (7) does not include a constant term. The modified model in Sect. 2.3, however, requires a model with a constant term. Therefore, we add a constant term $\mu$ in (7) and write it as

8 $$\begin{aligned} \varvec{S}\varvec{y}=\varvec{1}\mu + \varvec{\varepsilon } \end{aligned}$$

where $\varvec{S}=\varvec{I}_T-\varvec{C}$ with $\varvec{I}_T$ being a $T\times T$ identity matrix, $\varvec{1}$ is a $T\times 1$ vector of 1’s and $\mu$ is a scalar. If we assume $\varvec{\varepsilon }$ $\sim$ $\mathcal {N}(\varvec{0},\sigma ^2\varvec{I}_T)$, the parameters of the model can be estimated by maximizing the following log-likelihood function:

9 $$\begin{aligned} l({\theta })=-\frac{T}{2}\ln (2\pi )-\frac{T}{2}\ln \sigma ^2-\frac{(\varvec{S}\varvec{y}-\varvec{1}\mu )'(\varvec{S}\varvec{y}-\varvec{1}\mu )}{2\sigma ^2} \end{aligned}$$

where the parameter vector is $\varvec{\theta }=(\mu ,w_1,\dots ,w_M,\sigma ^2)'$. Equation (9) is generally maximized numerically. However, deriving the log-likelihood w.r.t $\mu$ we can obtain for it a conditional solution:

10 $$\begin{aligned} \hat{\mu }= & {} \varvec{1}'\varvec{S}\varvec{y}\nonumber \\= & {} \varvec{1}'\varvec{I}_T \varvec{y}-\varvec{1}'\varvec{C}\varvec{y}\\= & {} \sum \limits _{t=1}^T y_t - \sum \limits _{t=1}^T\tilde{y}_t=\sum \limits _{t=1}^T(y_t-\tilde{y}_t)\nonumber \end{aligned}$$

where $\tilde{y}_t=\sum \nolimits _{i<t} c_{t,i} y_i$ or $\tilde{y}_t=\sum \nolimits _{i\ne t} c_{t,i} y_i$ depending on the version of the model being used. The equation above shows that the constant term is a sum of deviations of $y_t$ from a linear combination of its values for all $t=1,\dots ,T$.^2^

### Regime-switching empirical similarity

Equation (8) in Sect. 2.2 is the specification of the model with a single constant. In this section, I present the ES model slightly modified in the spirit of regime-switching models. Namely, we allow the parameters to vary across *K* different states. This is akin to an autoregression with different parameters conditional on the value of a state variable $\mathcal {S} = \{1,2,\dots ,K\}$. Likewise, our parameter vector $\varvec{\theta }_k=(\mu _k,w_{1,k},\dots ,w_{M,k})'$ will be different for $k = 1,\dots , K$. Note that for the purpose of this study we assume error variance to be constant across states. The ES model with regime switching can then be written as follows:

11 $$\begin{aligned} y_t = {\left\{ \begin{array}{ll} \mu _1 + \sum \limits _{i\ne t} \psi _{t,i}^{(1)} y_i+\varepsilon _t \text { for } \mathcal {S}_t=1\\ \mu _2 + \sum \limits _{i\ne t} \psi _{t,i}^{(2)} y_i+\varepsilon _t \text { for } \mathcal {S}_t=2\\ \vdots \\ \mu _K + \sum \limits _{i \ne t} \psi _{t,i}^{(K)} y_i+\varepsilon _t \text { for } \mathcal {S}_t=K\\ \end{array}\right. } \end{aligned}$$

where $\mu _k$ is a state-specific constant term and $\psi _{t,i}^{(k)}$ is a state-specific similarity measure as described in Sect. 2.2.

To complete the specification of the model, we have to provide a state-generating process for the discrete variable $\mathcal {S}_t$. Let $\varvec{\gamma }$ be a $K\times 1$ vector with a single element being 1 and the rest equal to 0. The *k*th element of the vector $\varvec{\gamma }_t$ equal to 1 indicates $\mathcal {S}_t=k$, and the similarity model is governed by the parameters specific to the state *k*. In reality, $\gamma _{k,t}$ is an unobservable random variable and takes the value of 1 with probability $\pi _k$ or 0 otherwise. In regime-switching time series literature, $\varvec{\gamma }_t$ is assumed to evolve as a first-order Markov chain across *t*. However, for simplicity we assume its elements to be *i*.*i*.*d* random variables with expectations $E(\gamma _{k,t})=\pi _{k}$ which is also the probability $P(\mathcal {S}_t=k)$. Hence, $E(\varvec{\gamma }_t)=(\pi _{k})'_{k\le K}$ represents a probability distribution over the values of the state variable $\mathcal {S}$ at a given *t*. The assumption of an i.i.d state variable is also conditioned by the fact that we focus on the unordered version of the ES model which is by nature incompatible with Markov chains.

Given all the components of the model, one can write the likelihood function for maximum likelihood estimation. Let $f_k(y_t|\varvec{\theta }_k;\mathcal {F}_t$) be a conditional PDF of $y_t$ with an information set $\mathcal {F}_t=\{y_i|i\ne t;i,t=1,\dots ,T\}$. One can collect conditional PDF’s in a vector $\varvec{f}_t=(f_k)_{k\le K}$. A sample likelihood function is then given as

12 $$\begin{aligned} L(\varvec{y}|\Theta )=\prod _{t=1}^{T}\varvec{f}_t'\varvec{\gamma }_t \end{aligned}$$

where $\varvec{y}$ is a $T\times 1$ vector collecting the samples of *y* and $\Theta$ is the parameter set. Equation (12) shows that the likelihood is simply the mixture of conditional PDF’s. If we assume normal errors, then the log-likelihood function is given as

13 $$\begin{aligned} \ell (\Theta |\varvec{y}) = \sum \limits _{t=1}^T \ln \left\{ \sum \limits _{k=1}^K \gamma _{k,t}(2\pi \sigma ^2)^{-\frac{1}{2}}\exp \left( -\frac{\nu _{k,t}^2}{2\sigma ^2}\right) \right\} \end{aligned}$$

where $$\nu _{k,t} = y_t-\mu _k-\sum \nolimits _{i\ne t}\psi ^{(k)}_{t,i}y_i$$.

### Estimation

Equation (13) cannot be directly maximized to estimate the parameters in the model (11).

The reason is obviously the presence of the unobserved variable $\varvec{\gamma _t}$. Instead, we will have to replace it by the estimate $\hat{\varvec{\gamma _t}}$ of the expectation $E(\varvec{\gamma _t})=(\pi _k)'_{k\le K}$ which can be calculated using some filtering algorithm. For each *t*, we obtain the estimates $\hat{\varvec{\gamma }_t}$ by invoking the Bayes’s updating rule:

14 $$\begin{aligned} \hat{\varvec{\gamma }_t}=\frac{\varvec{f}_t\odot \varvec{\gamma }_p}{\varvec{1}'(\varvec{f}_t\odot \varvec{\gamma }_p)} \end{aligned}$$

where $\varvec{f}_t$ is defined as in the previous section, $\varvec{\gamma }_p$ is a $K\times 1$ vector of proportions representing the prior distribution of states and $\odot$ is an elementwise product. This is an application of the expectation maximization (EM) algorithm. The EM algorithm is a general iterative estimation technique designed for parameter estimation in models with unobservable stochastic variables. The way of implementation of the algorithm in this study relates the suggested model to the class of finite mixture models (see Dempster et al. 1977, pp. 15–17). The estimation procedure is summarized through the following iterative process.

1. **Initialization**: Initialize the parameter vector as $\hat{\varvec{\theta }}=\varvec{\theta }_0$.
2. **Filtering**: Using (14) and $\hat{\varvec{\theta }}$ compute the matrix $\hat{\varvec{\Gamma }}$ and update the prior as $\varvec{\gamma }_p=T^{-1}\hat{\varvec{\Gamma }}\varvec{1}$. $\hat{\varvec{\Gamma }}$ is a $K\times T$ matrix with columns being $\hat{\varvec{\gamma }}_t$ for $t=1,\dots ,T$.
3. **Optimization**: Maximize the log-likelihood function using $\hat{\varvec{\Gamma }}$ from step 2 and set the value of $\hat{\varvec{\theta }}$ equal to the optimized value from this step.
4. Repeat steps 2 and 3 until convergence criteria are satisfied.

It is important to start the estimation procedure with a reasonable initial value for the parameter vector due to possibility of multiple local maxima. I set the initial values equal to the result of the optimization problem:

15 $$\begin{aligned} \varvec{\theta }_0= & {} \underset{\theta }{{\text {argmin}}} \left( \sum \limits _{k=1}^K\gamma _{p,k}(\varvec{S}_k\varvec{y}-\varvec{1}\varvec{\mu }_k)\right) '\left( \sum \limits _{k=1}^K\gamma _{p,k}(\varvec{S}_k\varvec{y}-\varvec{1}\varvec{\mu }_k)\right) \\ \nonumber&s.t. \varvec{1}'\varvec{\gamma }_p=1\\&\gamma _{p,k}\ge 0, \forall k.\nonumber \end{aligned}$$

The estimate $\tilde{\varvec{\gamma }}_p$ should then contain the relative proportions of observations with distinct means and serve as a good starting point for a prior distribution of states. It is rather obvious that the EM procedure described above should provide improvement over $\varvec{\theta }_0$ because the elements of $\hat{\varvec{\Gamma }}'\varvec{\mu }$ have more degrees of freedom than those of $\varvec{1}\varvec{\gamma }_p'\varvec{\mu }$ due to filtering.

## Comparative simulation study

The aim of the simulation study is to compare the accuracy of point forecasts of the ES and baseline models. Replications of simulated data will be used to estimate the following set of models:

i. *First-order autoregression*
ii. *Markov-switching autoregressions*. I will use two-state and three-state Markov-switching autoregressions as *baseline models* and denote them, respectively, as MS(2)AR(1) and MS(3)AR(1).
iii. ES model as specified in (4). However, we will only focus on the case $i\ne t$, because it uses the whole dataset to predict individual observations.
iv. ES model modified to have mixed constant terms. The focus here will as well be on the version of the model with $i\ne t$.

The following sections describe the simulation design, data-generating processes and simulation results.

### Simulation design

I will generate a fixed data set ${D}^*=(y_t,\varvec{x}_t)_{t\le T}$ and a collection of data sets $\varvec{D}=\{{D}_i,i=1,\dots ,N\}$, where $N=1000$. Each dataset ${D}_i$ will be of length 500. The models will be estimated on all elements of ${\varvec{D}}$ and the estimated models will be used to generate a series of prediction errors $PE_{\mathcal {D}^*} = T^{-1}\sum \nolimits _{t\le T}(\tilde{y}^{(i)}_{t,j}-\hat{y}_t)^2$, where $\tilde{y}^{(i)}_{t,j}$ is a prediction of the model *k* estimated using data *i* in $\varvec{D}$ and $\hat{y}_t$ is a true prediction for a datapoint *t*. With a given series on $PE_{\mathcal {D}^*}$, we will then estimate the expected prediction error as

16 $$\begin{aligned} \hat{E}(PE_{\mathcal {D}^*}) = \frac{1}{N}\sum _{i=1}^{N}\left( T^{-1}\sum \limits _{t\le T}(\tilde{y}^{(i)}_{t,j}-\hat{y}_t)^2\right) \end{aligned}$$

for each model *j*. Here, as mentioned above, $\tilde{y}_{t,j}$ and $\hat{y}_t$ are, respectively, estimated and true predictions for the elements in data $\mathcal {D}^*$. Knowledge of $\hat{y}_t$ allows us to calculate the expected prediction error given by (16) without contaminating it with an irreducible error due to error variance.

#### Data-generating process and parameter values

To define the data-generating process (DGP), I use a regime-switching AR(p) model. According to a regime-switching AR(p) model, the sequence $\{y_t\}_{t=1}^T$ is generated according to

17 $$\begin{aligned} y_t=\mu _k+\sum \limits _{l=1}^{L}\alpha y_{t-l}+\epsilon _t \end{aligned}$$

where $\epsilon _t\sim N(0,\sigma ^2)$, $\alpha$ is the autoregressive parameter and $\mu _k$ is a regime-dependent constant term. For the purpose of this study, I assume only constant terms to be affected by a regime change. I set the lag order *L* equal to 1 for our DGP. Given that there are *K* states, such that $\mathcal {S}=\{1,\dots ,K\}$, values of $\mathcal {S}$ indicate the state of the system prevalent at *t*. I assume that $\mathcal {S}_t$ follows a first-order discrete-state Markov process with a transition matrix

18 $$\begin{aligned} \varvec{P}=\begin{pmatrix} p_{1,1}&{}\quad p_{1,2}&{}\quad \dots &{}\quad p_{1,K}\\ p_{2,1}&{}\quad p_{2,2}&{}\quad \dots &{}\quad p_{2,K}\\ \vdots &{}\quad \dots &{}\quad \ddots &{}\quad \vdots \\ p_{k,1}&{}\quad p_{k,2}&{}\quad \dots &{}\quad p_{K,K}\\ \end{pmatrix} \end{aligned}$$

where $\sum \nolimits _{h=1}^{K}p_{k,h}=1$, for $k=1,\dots ,K$. I will consider the cases $K=2$ and $K=3$ as these are empirically most relevant. In the beginning of the main section, it was mentioned that a baseline set of models includes two- and three-state Markov-switching autoregressions. Hence, with data generated through (17) with $K=\{2,3\}$ we would estimate correct specifications with MS(2)AR(p) and MS(3)AR(p) as our baseline models. In order to introduce a specification error to all models in the set, we allow regime switching between $K=2$ and $K=3$ versions of our DGP in (17). Therefore, the final DGP for simulating data ends up being a mixture of two- and three-state Markov-switching autoregressions. I let these mixture probabilities also follow a first-order Markov process, though with different levels of state persistence.

If one gives it a consideration, in reality systems with alternating number of states are possible. For instance, in financial markets prices of tradable products, on the one hand, tend to move sideways before trending in particular direction and eventually break in an opposite direction. On the other hand, they can evolve in a certain price corridor over an extended period with bounds of the corridor serving as points of reversal into the opposite regime.

For our DGP, I set $p=1$ for the sake of simplicity; hence, we deal with Markov-switching first-order autoregressions. The two components of the DGP with $K=2$ and $K=3$ have the following transition matrices:

19 $$\begin{aligned} \begin{array}{cc} P_{K=2}=\begin{pmatrix} .90 &{}\quad .10\\ .10 &{}\quad .90 \end{pmatrix}, &{} P_{K=3}=\begin{pmatrix} .90 &{}\quad .1 &{}\quad .00\\ .05 &{}\quad .85 &{}\quad .10\\ .00 &{}\quad .20 &{}\quad .80 \end{pmatrix} \end{array} \end{aligned}$$

In $P_{K=2}$, states are highly persistent. Values for $P_{K=3}$ are chosen as somewhat representative of business cycle literature where three states of economy are identified and stagnation and recessions are less persistent than expansions. Also, the probability of transition from recession directly to expansion is almost zero.

The coefficients assumed to be influenced by a regime change are the constant terms in (17). For a two-state component of the DGP, they will be set as $\mu _{K=2}=\{2,-1.5\}$ and for a three-state component as $\mu _{K=2}=\{3,0,-2\}$. Thus, respectively, for $K=2$ and $K=3$, these values could be thought of as qualitatively representing “expansion and contraction” and “expansion, stagnation and contraction” states of output growth in the business cycle literature.

I will compare the models in high and low autoregressive dynamics environment. Accordingly, the autoregressive coefficient will take the values $\alpha _\mathrm{low}=\{0.10;0.20;0.30\}$ and $\alpha _\mathrm{high}=\{0.70;0.80;0.90\}$. As described above, DGP will simply be a mixture of two- and three-state Markov-switching autoregressions with parameters given above. I let the mixture probabilities follow a Markovian process with a transition matrix

20 $$\begin{aligned} \mathcal {P}=\begin{pmatrix} z_{11}&{}\quad 1-z_{11}\\ 1-z_{22}&{}\quad z_{22} \end{pmatrix} \end{aligned}$$

and set of persistence probabilities as $z_{11} = z_{22} = 0.90$.

### Simulation results

To reiterate, our simulation study aims at investigating the predictive performance of the ES models in comparison with a set of alternatives. For this purpose, the data-generating process described in the previous section has been used to simulate data in low and high autocorrelation environments.

**Table 1:** Expected prediction error

$\alpha$	AR(1)	ES	MMES(2)	MMES(3)	MS(2)AR(1)	MS(3)AR(1)
(a)						
.1000	1.6599	1.6335	1.2285	1.3511	0.7679	0.5971
	(0.0230)	(0.0167)	(0.0842)	(0.3043)	(0.1052)	(0.0816)
.2000	1.9356	1.9212	1.2503	1.4054	0.8698	0.6804
	(0.0220)	(0.0171)	(0.0751)	(0.3319)	(0.1368)	(0.1149)
.3000	1.7873	1.7244	1.2490	1.3876	0.8572	0.6785
	(0.0277)	(0.0287)	(0.0898)	(0.3039)	(0.0933)	(0.0854)
(*b*)						
.7000	2.0567	2.1152	1.4888	1.8676	2.1481	1.9299
	(0.1493)	(0.1263)	(0.3100)	(0.6009)	(0.5294)	(0.4183)
.8000	2.6848	2.9077	1.6883	2.5722	3.9544	4.5662
	(0.2050)	(0.1901)	(0.4777)	(0.9411)	(1.2180)	(1.3611)
.9000	3.8962	4.2258	2.7908	3.7787	8.1846	7.8300
	(0.7982)	(0.6513)	(1.1200)	(1.6460)	(2.7049)	(3.1827)

In parentheses, standard deviations over replications are given

Table 1 contains the prediction error given in (16) for all six models alongside the standard errors in brackets calculated over the replications. All autoregressive models were estimated with lag order of 1. Similarly, first lag of the dependent variable was used as a feature in ES models. The column name MMES stands for mixed-mean empirical similarity.

The panel (a) in Table 1 contains prediction errors of six models in low autocorrelation data. As the highlighted figures show, Markov-switching AR(1) model with three states demonstrated the least prediction error. Nevertheless, it can be noted that MMES models definitely provide improvement over the predictive powers of AR(1) and the standard ES model. In panel (b), where results with higher autocorrelation coefficients are provided, the situation is rather different. Among six models, MMES(2) demonstrates the lowest prediction error followed by MMES(3) across all values of the autocorrelation coefficient.

The pattern in the results is rather unsurprising. With lower autocorrelation, the data contain less structure with dynamics being driven mostly through regime change in constant terms. Consequently, a relatively simpler model like Markov-switching autoregression would suffice to capture most of the movements in the target variable. In contrast, with larger autocorrelation coefficients, the data acquire richer dynamics: higher persistence coupled with sudden changes in the number of states leading to abrupt shifts in the mean of the process. Particularly, in the latter case, performance of MS autoregressions suffers because their specification assumes some stickiness in regime generation. However, under uncertainty about the regime generation, MMES model with an assumption of i.i.d state variable turns out to be more suitable.

## Empirical application: US GDP growth

In practice, while working with series potentially subject to structural changes, one usually possesses limited information about the nature of those. As long as this is the case, results of the simulation study indicate that ES models with regime-switching provide better predictions. In this section, we compare the empirical fit of models discussed above using data on economic series with an unknown regime-switching mechanism with potentially multiple regimes.

### Data and discussion

The seminal paper by Hamilton (1996) had set the start of a huge interest in the identification of business cycle phases in macroeconomic research. In particular, using Hamilton’s methodology, a number of subsequent research (i.e., Goodwin 1993; Filardo 2010; Chauvet et al. 2003; Billio and Casarin 2010; Golosnoy and Hogrefe 2013) focused on dating of turning points in the economic activity.

**Fig. 1:**
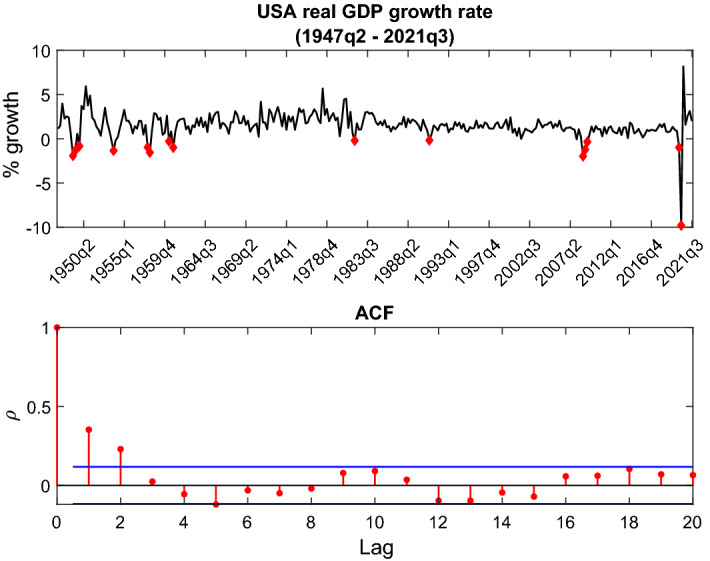
US quarterly real GDP growth rate

Although the academic research mainly focused on dating troughs and peaks due to their practical relevance, theoretically there are intermediate phases to the business cycle with characteristic behavior of macroeconomic indicators. The first panel of Fig. 1 plots the time series of growth rates of real gross domestic product of the USA through the period from the first quarter of 1947 to the second quarter of 2021. The growth rates were calculated using seasonally adjusted constant prices GDP series obtained from the Federal Reserve Bank of St. Louis database.

The points highlighted with diamonds mark the troughs as identified by the National Bureau of Economic Research (NBER). In the post-war period up until late 1980s, output growth fluctuated in a larger corridor with clear periods of low, medium and high growth. With the onset of the period of lower volatility of growth, also dubbed as the period of Great Moderation, high-growth periods, as they have been known, became nonexistent. With the lockdowns following the outbreak of COVID-19 pandemic, the high volatility of growth seems to have made a return. This pattern of behavior in the given series is loosely in the spirit of the DGP described in the simulation section. Therefore, the models from Sect. 3 will be estimated using these data in order to compare their explanatory power. The autocorrelation of the series decays quite fast (panel 2, Fig. 1), so we use a lag order of one for all models.

### In-sample comparison

As the aim of the paper is a comparison of the empirical power of models, we first discuss the in-sample fit. The specifications of the models described in the introduction of Sect. 3 were estimated using the real GDP growth data. Table 6 in Appendix contains the estimation results for all six models. A column corresponding to each model holds subcolumns with (i) full sample, (ii) subsample 1 and (iii) subsample 2 estimation results. Data were divided into subsamples at the fourth quarter of 1984 with a view to separate periods with vivid volatility differences (Fig. 1). Accordingly, the first subsample includes the period from the second quarter of 1947 to the fourth quarter of 1984 and the second subsample includes the rest except for the start of the pandemic.

As can be seen, only the constant terms were allowed to change across regimes. The constant terms of the regime-switching models indicate that there is clearly more than one state to the growth series. Nevertheless, results also indicate that three might be the maximum number of states. Although we have to perceive the standard errors with a caution due to their approximative nature, the coefficient estimates of MMES(3) model for “stagnation” state are not significant. The same result is confirmed by the MS3 model only for the subsample 1. It should be noted that the magnitudes of the constant terms of MS and MMES models are not directly comparable which is why they were reported in separate rows. However, one can calculate the unconditional means for MS models through $\hat{c}_j/(1-\hat{\alpha })$ for $j=1,\dots ,K$ to compare them with those of MMES models. Hence, for instance, “trough” coefficient (in subsample 1) $\hat{\mu }_1=-1.35$ of MMES(2) is comparable to the unconditional mean $-.85/(1-.45)\approx -1.54$ of the same state of MS(2) model.

In the case of full sample, an analogous comparison for the “peak” state between the same models reveals quite a big difference of approximately .95, meaning that MMES(2) model possibly underestimated the magnitude of the peaks in high growth period. The same pattern persists in subsamples as well. On the other hand, a similar comparison between MMES(3) and MS(3) models shows that “trough” coefficients in the former are much larger than in the latter in subsamples, whereas their “peak” coefficients are very close. Thus, it seems that the biggest difference in predictions of MS(3) and MMES(3) models may be attributable to the difference in their prediction of troughs, whereas the source of biggest difference between MS(2) and MMES(2) might be predictions of peaks. Nevertheless, as full-sample results show, inclusion of the COVID period significantly alters the estimation results relative to subsamples. Namely, explanatory power of all six models drastically reduces as indicated by the error variance estimates and the model fit criteria.

A usual practice in empirical research with MS models is to report regime persistence parameters. However, due to i.i.d specification of regime probabilities in MMES models, estimated proportions $\hat{\pi }_1,\dots ,\hat{\pi }_K$ for each state were reported. Although, in general, estimated relative frequencies of states are comparable between MS and MMES models, there are occasionally quite significant numerical differences. Namely, when compared to MS(2) model, MMES(2) model predicts extreme observations on the lower end more often, which possibly explains the above-mentioned difference of .95 in unconditional means for peak state. In contrast, MMES(3) predicts the peak state less often and weights stagnation state more heavily as compared to MS(3).

**Table 2:** In-sample prediction for extreme observations

	AR(1)	ES	MMES(2)	MMES(3)	MS(2)AR(1)	MS(3)AR(1)
MSE	12.2301	11.9964	4.9886	4.6822	7.6357	5.6055

**Fig. 2:**
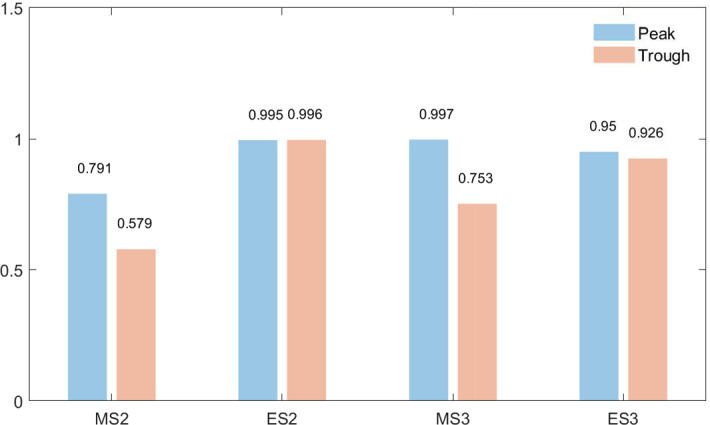
State prediction scores for extreme observations

Alongside the parameter estimates, overall measures of fit such as MSE, MAE and AIC were also reported. It turns out for all models it is uniformly the case that the subsample 2 was an easier sample to fit, perhaps due to a lower variance. Other than that, MMES models demonstrate a better overall in-sample fit. Probably, the ability to pick most relevant observations through an exponential similarity function coupled with a regime-switching constant term allows MMES models to better specialize in fitting individual observations. Table 2 and Fig. 2 summarize some evidence on a better propensity of MMES models to predict more extreme observations. In the current context, an observation $y_t$ is considered extreme if $y_t > c_{.95}$ or $y_t < c_{.05}$, where $c_{.05}$ and $c_{.95}$ are, respectively, $5\%$ and $95\%$ quantiles of the target variable. Figure 2 plots the state prediction scores (SPS) calculated as in (21) for each model.

21 $$\begin{aligned} SPS_{k}= & {} \frac{\sum ^T_{t=1}\hat{P}(\mathcal {S}_t=k|\mathcal {F}_t)I_{A_k}}{\sum ^T_{t=1}I_{A_k}} \end{aligned}$$

where $\mathcal {S} =$ {*‘peak’*,*‘trough’*}, *I* is an indicator function with $A_k=\{y_t|y_t\le c_{.05}\}$ for $k=$*‘trough’* and $A_k=\{y_t|y_t\ge c_{.95}\}$ for $k=$*‘peak’*. Both MMES models demonstrate much lower mean squared error values in comparison with the alternatives. In addition, both models seem to attain scores at least as good as those of baseline models in assigning extreme observations into peak and trough states (Fig. 2).

### Out-sample comparison: Recessions

In the previous section, we have stressed the ability of MMES models to better capture the extreme observations in sample. This section presents the results on an exercise of out-sample prediction of selected extreme points.

The selected quarters and corresponding values of percentage changes in US real GDP are given in Table 3.

**Table 3:** Recession quarters

Quarters	Real GDP, % change
1949q1	$-$ 1.92
1949q2	$-$ 1.35
1949q4	$-$ 0.83
1953q4	$-$ 1.34
1957q4	$-$ 0.97
1958q1	$-$ 1.55
1960q2	$-$ 0.29
1960q4	$-$ 0.99
1982q1	$-$ 0.20
1990q4	$-$ 0.17
2008q4	$-$ 1.97
2009q1	$-$ 1.22
2009q2	$-$ 0.34
2020q1	$-$ 0.99
2020q2	$-$ 9.79

**Table 4:** MSE for predictions of recession quarters in Table 3

	AR(1)	ES	MME(2)	MMES(3)	MS(2)AR(1)	MS(3)AR(1)
25%	4.3851	5.2104	0.4309	0.9291	2.9936	1.2883
50%	12.4707	12.9232	3.1914	5.5687	9.9903	7.1502
75%	13.5275	14.2531	4.6295	6.2701	10.8805	8.0965
100%	18.2872	19.0582	8.1844	9.0480	15.4336	11.9599

As mentioned in previous sections, all models were estimated with lag order of one. Dropping only selected points out of the estimation would be inconsistent with first lag of the target variable serving as explanatory variable. Therefore, we also exclude observations immediately preceding the selected dates. Moreover, after the estimation, excluded data are inserted back into their original positions to preserve time ordering in data.

Table 4 presents MSE values with different portions of outliers left out of estimation. As the outliers leave the data, performance of all models declines as there is progressively less in-sample information about extreme points. Nevertheless, in all scenarios, MMES models demonstrate less error in predicting the extreme observations.

Although MSEs for point predictions at par value favor the MMES models more, it is worth to test the significance of the difference between model predictions. For this purpose, we employ the Diebold–Mariano (DM) test^3^ and compare ES, MMES(2) and MMES(3) models to baseline models AR(1), MS(2)AR(1) and MS(3)AR(1), thus conducting nine comparisons in total.

In each test, the null hypothesis reads as “*predictions by the baseline model are more accurate*.” The DM test uses the statistic $\bar{d} = P^{-1}\sum _{\tau = 1}^{P} \left[ g(e_{i\tau }) - g(e_{j\tau })\right]$ where $e_{i\tau }$ and $e_{j\tau }$ are prediction errors from candidate models *i* and *j*, *g* is a loss function on the prediction error and *P* is the number of points being predicted. The results of DM test are influenced by the choice of the loss function. Common candidates for *g* are square or absolute values of prediction errors. The quadratic loss function is much more sensitive to extreme values in *e*. Therefore, Table 5 contains the test results for nine comparisons of predictions of recession quarters given in Table 3 using both (a) quadratic and (b) absolute value loss functions. In panel (a), where results with quadratic loss function are presented, the null hypothesis is uniformly rejected for MMES(2) in all comparisons at 5$\%$ significance level. Although MMES(3) delivered more accurate predictions than AR(1) and MS(2)AR(1), they are not significantly better than those of MS(3)AR(1). With an absolute value loss function (panel b) test statistics increase in values raising the significance of MMES models. Notably, MMES(3) becomes significant vis-a-vis the baseline. A quadratic *g* shoots up with a large $e_{i\tau }$, thereby reducing the power of the DM test to reject the null hypothesis. On the other hand, absolute value *g* does not allow a single $e_{i\tau }$ to dominate the measure of predictive performance of a model, which might have been reflected in the comparison of MMES(3) against MS(3)AR(1) in panel (b).

**Table 5:** Test statistics for one-sided DM test

	AR(1)	MS(2)AR(1)	MS(3)AR(1)
(a)			
ES	0.6331	1.9040	2.1166
	(0.7367)	(0.9715)	(0.9829)
MMES(2)	$$-2.0732^{**}$$	$$-1.8939^{**}$$	$$-1.675^{**}$$
	(0.0190)	(0.0291)	(0.0469)
MMES(3)	$$-3.5145^{***}$$	$$-3.9548^{***}$$	$$-1.2453$$
	(0.0002)	(0.0000)	(0.1065)
(b)			
ES	$-$ 0.6580	1.9232	3.6537
	(0.2553)	(0.9728)	(0.9999)
MMES(2)	$$-6.9456^{***}$$	$$-5.3668^{***}$$	$$-3.7933^{***}$$
	(0.0000)	(0.0000)	(0.0001)
MMES(3)	$$-16.623^{***}$$	$$-6.847^{***}$$	$$-1.9994^{**}$$
	(0.0000)	(0.0000)	(0.0228)

*p* values are given in parentheses

## Conclusions

In the paper, I have revisited the empirical similarity model of Gilboa et al. (2006) and suggested a modification on the basis of an established methodology. In particular, regime change mechanism has been introduced into the data-generating process of the model. In its new form, the model relates to the class of regime-switching regression models. Namely, it has been argued that the modified model could be considered as an alternative to Markov-switching autoregressions. The latter, despite being designed to account for parameter changes, remains linear within a given state. On the contrary, the modified model unites the intrinsic nonlinearity of the original ES model with the capability of the regime change mechanism to accommodate changes in model parameters. This attempt to equip the ES model with a regime-switching mechanism can be justified at least with two arguments. First, the DGP of the ES model is quite general and can be applied in many contexts. Second, for processes with regime switching, generation of values for the state variable might be misspecified by Markov-switching models. In this case, MMES model with rather simpler assumptions on the behavior of a state variable might perform better.

The simulation exercise has made an attempt to compare the ES models with baseline alternatives under a data-generating process that involved a non-trivial regime change process. Results on the predictive power of the models show that with weak autocorrelation dynamics in data the modified ES model is superior only to models which do not involve any regime-switching behavior. As low autocorrelation implies less structure in data, it is rather obvious that intrinsic nonlinearity of ES impairs its external validity. Nevertheless, with data generated on the higher end of autocorrelation spectrum, the modified ES model delivers more accurate predictions.

One can name a few advantages to the suggested model. First, it brings versatility and can be applied as a universal model to contexts involving predictions. Second, the modified ES could be the model of preference if the process being modeled is subject to generating more extreme observations. Finally, even though the model is nonlinear, it can be very easily implemented as it does not involve complicated smoothing algorithms as in Markov-switching models. One could argue that simplicity comes at the cost of possibility of making inference on state probabilities and estimating the state durations. Nevertheless, if the objective is to obtain more accurate predictions under uncertainty about the true data-generating process, the modified ES could be the preferred model.

**Table 6:** Estimation results for all models: (i) full sample, (ii) subsample 1 and (iii) subsample 2

	AR			ES			MMES(2)			MMES(3)			MS(2)AR(1)			MS(3)AR(1)		
	(i)	(ii)	(iii)	(i)	(ii)	(iii)	(i)	(ii)	(iii)	(i)	(ii)	(iii)	(i)	(ii)	(iii)	(i)	(ii)	(iii)
$\hat{w}$				0.61	1.18	0.93	0.37	1.26	4.10	0.61	0.35	0.64						
				(0.03)	(0.05)	(0.09)	(0.28)	(0.43)	(1.19)	(0.29)	(0.25)	(0.33)						
$\hat{\alpha }$	0.25	0.35	0.37										0.26	0.45	0.55	0.51	0.27	0.51
	(0.06)	(0.08)	(0.08)										(0.06)	(0.12)	(0.09)	(0.05)	(0.11)	(0.11)
$\hat{c}_1$	1.15	0.58	0.39										1.39	$-$ 0.85	$-$ 0.91	$-$ 8.54	$-$ 1.09	$-$ 0.94
	(0.10)	(0.10)	(0.07)										(0.13)	(0.57)	(0.21)	(0.92)	(1.12)	(0.32)
$\hat{c}_2$													1.67	1.10	0.68	1.53	0.13	0.45
													(0.12)	(0.15)	(0.07)	(0.11)	(0.30)	(0.10)
$\hat{c}_3$																8.67	1.30	0.83
																(0.92)	(0.15)	(0.10)
$\hat{\mu }_1$				1.53	0.89	0.63	$-$ 1.35	$-$ 1.30	$-$ 0.68	$-$ 1.85	$-$ 3.34	$-$ 2.24						
				(0.00)	(0.01)	(0.00)	(0.39)	(0.25)	(0.30)	(0.60)	(2.85)	(0.82)						
$\hat{\mu }_2$							1.05	0.84	0.57	0.20	$-$ 0.05	$-$ 0.03						
							(0.56)	(0.17)	(0.17)	(0.94)	(1.07)	(2.85)						
$\hat{\mu }_3$										1.99	1.66	1.37						
										(0.49)	(0.58)	(1.07)						
$\hat{\pi }_{1}$							0.40	0.38	0.41	0.10	0.06	0.04	0.49	0.11	0.04	0.06	0.06	0.02
$\hat{\pi }_{2}$							0.60	0.62	0.59	0.82	0.76	0.89	0.51	0.89	0.89	0.78	0.22	0.41
$\hat{\pi }_{3}$										0.08	0.18	0.07				0.16	0.73	0.51
$\hat{\sigma ^2}$	1.65	1.18	0.29	1.61	1.25	0.31	0.75	0.36	0.10	0.66	0.33	0.12	1.63	0.76	0.19	0.89	0.79	0.17
	(0.05)	(0.12)	(0.03)	(0.01)	(0.01)	(0.01)	(0.24)	(0.05)	(0.02)	(0.14)	(0.12)	(0.19)	(0.06)	(0.16)	(0.03)	(0.07)	(0.11)	(0.03)
MSE	1.66	1.19	0.30	1.61	1.25	0.31	0.78	0.36	0.10	0.67	0.33	0.12	1.64	0.61	0.18	0.92	0.68	0.15
MAE	0.77	0.87	0.41	0.77	0.89	0.40	0.37	0.46	0.22	0.48	0.45	0.29	0.77	0.64	0.34	0.71	0.69	0.31
AIC	157.30	32.37	− 159.69	83.38	37.49	− 155.61	− 65.03	− 143.70	− 313.61	− 139.15	− 152.83	− 277.85	156.41	− 54.20	− 203.88	− 0.83	− 32.59	− 222.59

Standard errors of MS and ES models were calculated by numerical approximation

## A Estimation output

See Table 6.

## Footnotes

1 Gilboa et al. (2006) have briefly discussed how to adopt the model to deal with time series in a way that resembles autoregression.

2 Note the similarity to the OLS estimator of a constant term in a simple linear regression $y_t=\beta _0+\beta x_t+\varepsilon _t$: $\hat{\beta }_0=\bar{y}-\hat{\beta }_1 \bar{x}=T^{-1}\sum \nolimits _{t=1}^T(y_t-\hat{y}_t)$

3 See Diebold and Mariano (2002) for a full discussion of the test.
